# Kappa accuracy of prototypical diagnosis and ICD-10 criteria for mental disorders: A cross-sectional study in a real-life setting

**DOI:** 10.1192/j.eurpsy.2022.434

**Published:** 2022-09-01

**Authors:** H.G.D. Rocha Neto, M. Cavalcanti, D. Telles Correia

**Affiliations:** 1 Universidade Federal do Rio de Janeiro, Instituto De Psiquiatria Da Ufrj, Rio de Janeiro, Brazil; 2 Universidade Federal do Rio de Janeiro, Centro De Ciências Da Saúde, Rio de Janeiro, Brazil; 3 Lisbon Medical University, Psychiatry, Lisbon, Portugal; 4 FACULDADE DE MEDICINA UNIVERSIDADE DE LISBOA, Psychiatry, Lisboa, Portugal

**Keywords:** reliability, psychiatry, diagnosis, validity

## Abstract

**Introduction:**

The use of “operational criteria” in DSM-III was proposed as a solution to low reliability among psychiatrist’s diagnosis. It is considered a turning point in the psychiatric classification and diagnostic process, furtherly adopted in ICD. However, the utility of using such criteria in everyday clinical practice is still not clear.

**Objectives:**

To measure agreement between prototypical and ICD-10 categorical diagnosis.

**Methods:**

In IPUB’s outpatient clinics, psychiatry residents work in a real-life clinical scenario, attending patients from Rio de Janeiro/RJ-Brazil. Although regularly trained in ICD criteria, it is not usual to check every criterion in their daily practice. Thus, patients are diagnosed with a prototype-based disorder, not necessarily strictly attached to ICD criteria. We propose a cross-sectional study, where psychiatry residents check their clinical diagnosis according to ICD criteria and compare its agreement with kappa statistics.

**Results:**

Three of thirty residents joined the study, providing diagnosis for 146 patients under their care. Forty-five diagnoses were obtained before and 51 after ICD-10 criterion application. Diagnoses were grouped under 8 groups (Organic, Schizophrenia Spectrum Disorders, Bipolar Affective Disorder, Depression, Anxiety-Related Disorders, Personality Disorders, Neurodevelopmental Disorders), and kappa agreement obtained using ICD-10 diagnosis as the gold standard against prototypical diagnoses. Overall kappa was 0.77 (IC - 0.69 - 0.85), ranging from 0.58 (Personality Disorders) to 0.91 (Schizophrenia Spectrum Disorder). These findings also were reflected as high sensibility, specificity, Positive Predictive, and Negative Predictive values in all groups.

**Conclusions:**

Prototypical diagnostic elaboration, while probably based on previously learned, but not applied operational criteria, was equivalent to diagnostic obtained through ICD-10 categories.

**Disclosure:**

No significant relationships.

